# The Use of Hematopoietic Stem Cells for Heart Failure: A Systematic Review

**DOI:** 10.3390/ijms25126634

**Published:** 2024-06-17

**Authors:** Jayant Seth, Sohat Sharma, Cameron J. Leong, Venkat Vaibhav, Pierce Nelson, Arveen Shokravi, Yuchen Luo, Daniel Shirvani, Zachary Laksman

**Affiliations:** 1 Department of Medicine, University of British Columbia, Vancouver, BC V6T 1Z3, Canada; jayant.seth@student.ubc.ca (J.S.); sohat410@student.ubc.ca (S.S.); camleong@student.ubc.ca (C.J.L.); shokravi@student.ubc.ca (A.S.); yluo074@student.ubc.ca (Y.L.); dshirv@student.ubc.ca (D.S.); 2 Schulich School of Medicine and Dentistry, Western University, London, ON N6A 5C1, Canada; vmanimur@uwo.ca (V.V.); pnelso4@uwo.ca (P.N.); 3 School of Biomedical Engineering, University of British Columbia, Vancouver, BC V6T 1Z3, Canada; 4 Centre for Heart Lung Innovation, St. Paul’s Hospital, Vancouver, BC V6Z 1Y6, Canada

**Keywords:** stem cell therapy, hematopoietic, heart failure, clinical, ischemic heart damage

## Abstract

The purpose of this review is to summarize the current understanding of the therapeutic effect of stem cell-based therapies, including hematopoietic stem cells, for the treatment of ischemic heart damage. Following PRISMA guidelines, we conducted electronic searches in MEDLINE, and EMBASE. We screened 592 studies, and included RCTs, observational studies, and cohort studies that examined the effect of hematopoietic stem cell therapy in adult patients with heart failure. Studies that involved pediatric patients, mesenchymal stem cell therapy, and non-heart failure (HF) studies were excluded from our review. Out of the 592 studies, 7 studies met our inclusion criteria. Overall, administration of hematopoietic stem cells (via intracoronary or myocardial infarct) led to positive cardiac outcomes such as improvements in pathological left-ventricular remodeling, perfusion following acute myocardial infarction, and NYHA symptom class. Additionally, combined death, rehospitalization for heart failure, and infarction were significantly lower in patients treated with bone marrow-derived hematopoietic stem cells. Our review demonstrates that hematopoietic stem cell administration can lead to positive cardiac outcomes for HF patients. Future studies should aim to increase female representation and non-ischemic HF patients.

## 1. Introduction

Heart failure (HF) is a complex clinical syndrome marked by a constellation of various signs and symptoms. These symptoms arise from impaired cardiac function or structure, encompassing systolic and diastolic dysfunction. The current therapeutic approach to long-term HF management includes risk and lifestyle modifications; medications for symptom control, prevention and reversal of cardiac remodeling; and various device or surgical interventions [1]. The rising prevalence of HF worldwide, particularly within an aging population, in conjunction with an approximate 24% lifetime risk of developing HF [2], highlights the profound impact of HF on mortality and morbidity. Notably, post HF hospitalization, there is a 42% mortality rate over five years [1]. Moreover, HF-related complications such as arrhythmia, sudden cardiac death, thromboembolic events, hepatic and renal complications, along with diminished quality of life, emphasize the need for further innovative solutions to effectively manage and mitigate its detrimental effects on patients’ health and well-being.

The American Heart Association (AHA) classifies heart failure by left ventricular ejection fraction (LVEF) [3]. HFrEF is defined by LVEF ≤ 40%. HFpEF is defined by LVEF ≥ 50% with spontaneous or provokable increased LV-filling pressures. Increased LV-filling pressures are indirectly indicated by elevated NT-pro-BNP or hemodynamic measurements showing diastolic dysfunction. HFmrEF is defined by LVEF ≤ 41–49% with evidence of spontaneous or provokable increased LV-filling pressures. HFimpEF is defined by previous LVEF ≤ 40% and a follow-up measurement of LVEF ≥ 40%. HF treatment includes both pharmacological and intervention-based approaches. Pharmacological management of HF also considers the NYHA classification [3].

There are several therapies for patients with HFrEF. In patients with HFrEF and NYHA class II to III symptoms, ANRis, ACEis, or ARBs are often indicated. Beta blockers are also considered in HFrEF patients with current or previous symptoms. MRAs are recommended in patients with HFrEF and NYHA class II to IV. SGLT2i are indicated in patients with symptomatic chronic HFrEF. Finally, hydralazine and isosorbide dinitrate are recommended in African American patients with HFrEF NYHA class III-IV. HF medications, while effective in reducing morbidity and mortality, are not curative and patients may require their use for extended periods of time. Interventional therapies for HFrEF include implantable cardioverter defibrillators (ICDs) and cardiac resynchronization therapies (CRTs). ICD is recommended in patients with nonischemic dilated cardiomyopathy (DCM) or ischemic heart disease that lasts at least 40 days post myocardial infarction with LVEF ≤ 35% and NYHA class II–III.

In patients with HFmrEF and HFimpHF, SGLT2i is recommended to decrease HF hospitalization and mortality. Among patients with current or previously symptomatic HFmrEF, beta blockers, ARNi/ACEi/ARBs, and MRAs are also recommended to reduce HF hospitalization and mortality. Pharmacological management of HFpEF involves careful consideration of blood pressure. Medications should be titrated to attain blood pressure targets. In addition, SGLT2i, MRAs, ARBs, and ARNIs are considered to reduce HF hospitalization and mortality.

The etiologies of HF can be broadly categorized into those with ischemic and non-ischemic origins; within North America and Europe ischemic causes are more prevalent [2]. The pathophysiologic changes in ischemic heart disease are related to changes in cardiac tissue, which is composed in part of cardiomyocytes. Cardiomyocytes are terminally differentiated and exhibit markedly limited capacity for regeneration [4]. This limitation is critical, as ischemic injury to the heart will lead to permanent loss of these cells [1,4]. Following myocardial infarction (MI), cardiac tissue remodels as cardiomyocytes are replaced by non-contractile connective tissue [4]. This remodeling coupled with limited ability for compensation results in cardiac function decline and heart failure [1,5]. In contrast to ischemic heart failure, non-ischemic heart failure encompasses a diverse array of etiologies, including but not limited to those with genetic, inflammatory, toxic, or infectious origins [2]. The pathophysiological mechanisms of non-ischemic HF are unique to their given etiology and therefore may manifest with different clinical presentations, natural histories, and different therapeutic strategies. Nonetheless, these pathophysiological mechanisms can result in changes to contractility and/or ventricular compliance [1], in part due to effects on structure and function of cardiomyocytes.

Currently, cell-therapy has become increasingly prominent due to a number of promising preliminary results. However, despite the increased interest from both patients and clinicians, invariably there are numerous regulations based on a public health precedent that must be met prior to the utility of cell therapy as treatment for cardiovascular conditions. In the United States of America, the US Food and Drug Administration is currently responsible for regulating cell-based therapy as tissue products. There are also several clinics that operate outside of FDA-approved clinical trials that offer cell-based therapy to patients with cardiovascular conditions such as heart failure, termed as COURS. These clinics have been known to cite the RIGHT to try act, which provides a list of criteria for the patients. These include that patients must have a life-threatening illness, have exhausted clinical treatments, or are unable to enroll in a clinical trial [6].

**Figure 1:**
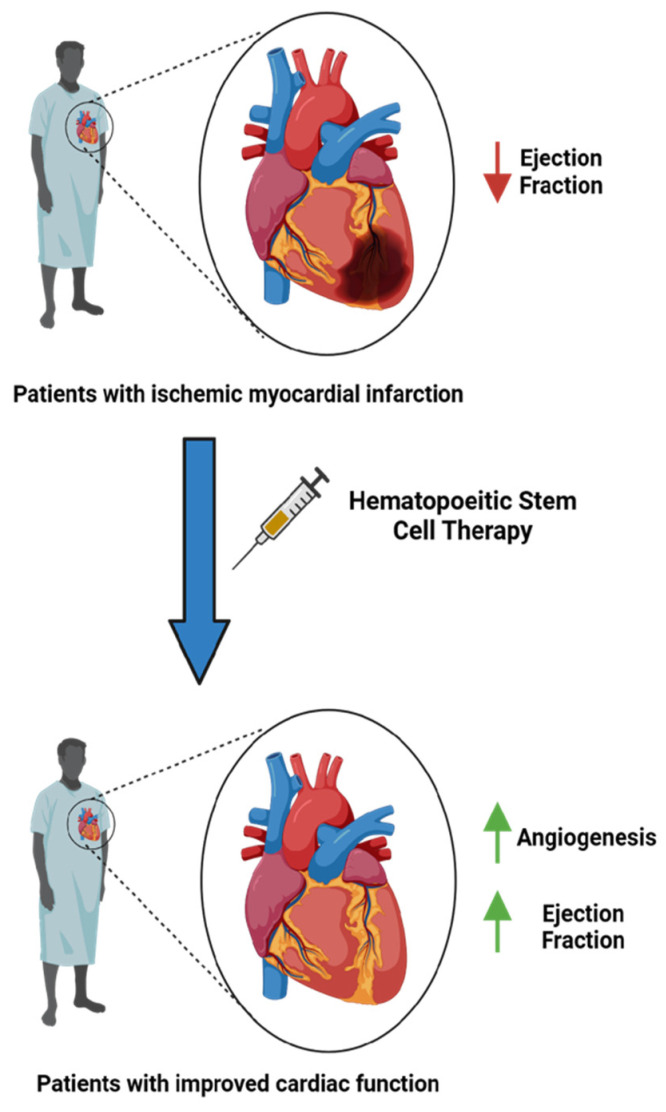
Hematopoietic stem cells may improve cardiac function and ejection fraction. Possible mechanisms of action include regeneration of cardiomyocytes, release of protective signaling molecules, trans-cell differentiation, and cell fusion with cardiomyocytes.

In contrast to cardiomyocytes, stem cells possess a remarkable capacity for self-renewal. Recent advancements in stem cell research aim to harness this reconstructive potential for cell therapy, especially in the context of heart failure [7]. The intricacies of the mechanisms by which these stem cells exert their regenerative effects on damaged cardiac tissue remain unclear. Older theories postulated that targeted stem cells integrate and differentiate into cardiomyocytes or supportive tissues, while newer hypotheses propose mechanisms which involve stem cells activating endogenous pathways that enhance cardiac performance [8]. Bolli et al. discuss that the beneficial effects of stem cell therapy on cardiac tissue are not due to the integration of transplanted cells directly into the myocardium, with eventual transition into cardiomyocytes, but rather through the release of signals and mediators that positively influence the host myocardium [8]. Although cardiac function improves post stem cell transplantation, there are limited signs of myogenesis, suggesting that the paracrine actions of stem cells may function through alternative mechanisms. These mechanisms may include reduced inflammation, extracellular remodeling, angiogenesis, or optimize function of remaining cardiomyocytes [8]. Research surrounding the therapeutic utility of bone marrow stem cells, including hematopoietic stem cells (HSCs) and mesenchymal stem cells (MSCs), continues to be investigated [9]. The literature has shown the efficacy of autologous bone marrow-derived stem cell transplantation in the reversal of diminished ejection fraction (Figure 1) [10].

Specifically, the therapeutic potential of HSCs in the context of HF may be described by multiple mechanisms including trans-cell differentiation as a result of the presence of cardiac transcription factors, cytokine-mediated repair, and cell fusion between cardiac myocytes and transplanted hematopoietic stem cells. Additionally, HSCs express a variety of cytokines and factors such as platelet-derived growth-factors, VEGF and insulin-like growth factor that promote myocardial regeneration through angiogenesis and cardiomyocyte stimulation [11].

A 2021 review has shown MSCs’ utility in cardiac regeneration [12], but to our knowledge, there is no recent review focusing on the efficacy of HSCs in this regard. The purpose of this review is to summarize the current understanding of the potential therapeutic effect of hematopoietic stem cells for the treatment of heart failure. We will assess treatment efficacy from a clinical perspective, and discuss changes in ejection fraction, laboratory markers such as NT-pro-BNP, and functional metrics including New York Heart Association (NYHA) classification. Additionally, we will evaluate the safety profile and post-therapy follow-up, and comment on hospital readmission and major cardiac events during this period. We aim to summarize and add to the current perspective on the clinical efficacy and safety of HSCs for heart failure with a major focus on ischemic cardiomyopathy.

## 2. Materials and Methods

### 2.1. Search Strategy

This review was directed following the 2020 Preferred Reporting Items for Systematic Reviews and Meta-Analyses (PRISMA) guidelines. The research question was developed using the PICO framework (Table 1). Electronic searches were conducted in MEDLINE and EMBASE with keywords: “hematopoietic stem cells” and “cardiomyopathy” from database inception to December 2023.

The inclusion criteria were all primary randomized controlled trials or cohort studies published in English that examined the effect of HSC therapy in adult patients with heart failure. The exclusion criteria were papers that involved pediatric patients, MSC therapy, and non-HF studies. Abstracts, editorials, case reports, and reviews were also excluded.

All references were uploaded to Covidence and were electronically merged to remove duplicates. Two authors individually reviewed each study to determine their inclusion or exclusion. The data extracted from each study were the following: study design, country in which the study was conducted, stem cell therapy and placebo sample sizes, and proportion of male patients. Additionally, the following algorithm characteristics were also extracted from each study: HF etiology and type (HFrEF/HFpEF), NYHA class, peak VO2 before and after therapy, ejection fraction (EF) before and after therapy, duration of follow-up appointments, and notable comorbidities in the patient population. Six reviewers (CL, VV, PN, AS, YC, DS) conducted data extraction and a consensus was reached for any conflicts. Any conflicts were resolved by SS.

### 2.2. Study Selection

**Figure 2:**
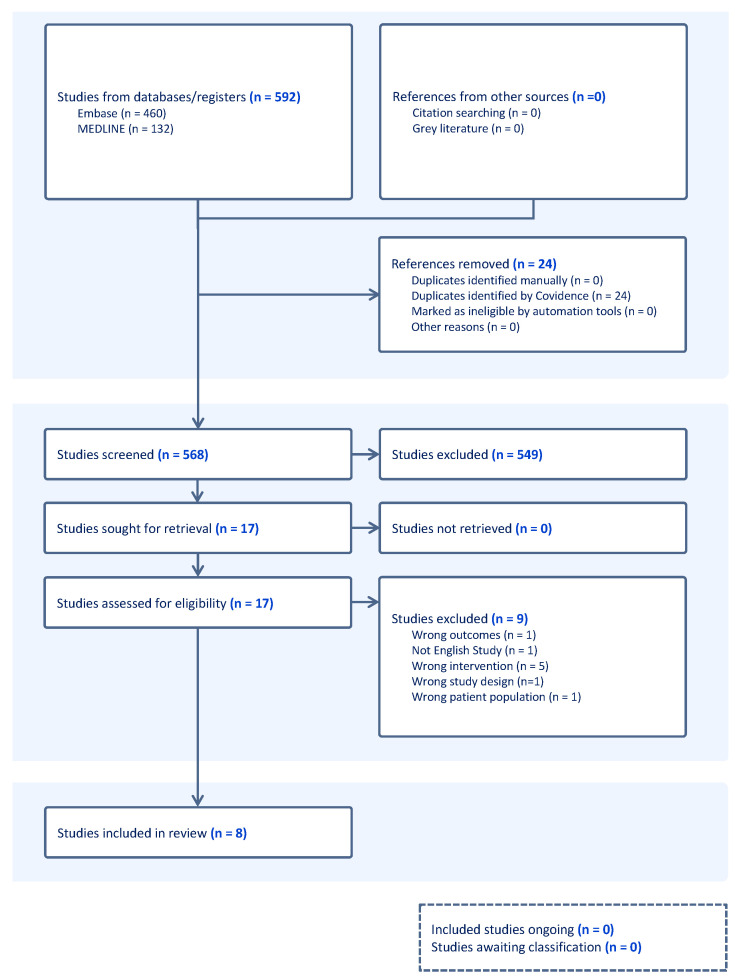
PRISMA flow diagram.

A total of 592 studies were uploaded onto Covidence for screening from the literature search. Of these, 565 studies were identified after duplicates were removed. Altogether, 549 abstracts were deemed irrelevant and 14 studies were examined for full-text review. Of these, 8 studies were included for data extraction. The study selection process is illustrated in Figure 2 below.

## 3. Results

### 3.1. Study Design

Due to the heterogeneity of the included studies, a meta-analysis was not feasible. The details of the included studies are shown in Table 2, including study designs and characteristics. A total of 71.4% (5/7) of the studies were from Europe (Table 2). A total of 28.6% (2.7) of the studies had a sample size greater than 50. A total of 57.14% (4/7) of our studies were randomized control trials, 1 was a prospective open-labeled control trial, 1 was a prospective cohort study, and 1 was a randomized control trial. The treatment for our studies was a mixture of HSCs and bone marrow stem cells. A total of 57.41% (4/7) of our studies looked only at HSCs as an intervention for heart failure, whereas the other 3 examined the use of bone marrow stem cells in general. All of our studies examined ischemic heart failure and non-ischemic heart failure and the outcomes that we examined included changes in ejection fraction, major adverse cardiovascular outcomes, and hospitalizations. It should be noted that the majority of the patients in the selected studies did have ischemic heart failure.

### 3.2. Impact of BMSC on LVEF and Myocardial Perfusion

Manginas et al. demonstrated that intracoronary administration of selected CD133+ and CD133 CD34+ progenitor cells resulted in long-term decreases in left ventricular end-diastolic and end-systolic volume and also showcased an improvement in ejection fraction (from 27.2 ± 6.2% to 29.7% ± 7.3%, *p* = 0.016). Moreover, patients undergoing BMSC intracoronary administration had significantly better apex perfusion ratio % at baseline (32.4 ± 7.1) compared to at 12-month follow-up (44.0 ± 10.3%), *p* ≤ 0.001 and anterior perfusion ratio %: baseline = 39.2% ± 9.0% to 47.3 ± 12.7%, *p* = 0.005]. Overall, these results indicate that intracoronary administration of these selected bone marrow mononuclear stem cells could be beneficial in improvements in pathological left-ventricular remodeling and perfusion following acute myocardial infarction [13].

Assmus et al. randomized 204 patients also with acute myocardial infarction (AMI) into receiving either intracoronary-administered bone marrow-derived progenitor cells (BMC) or placebo. This study did not show significant changes in MRI-derived left-ventricular ejection fraction; albeit, LVEF tended to be higher in treatment (50.1% [95% CI, 46.5 to 53.7] versus 43.6% [95% CI, 40.4 to 46.8], P0.14). However, angiography-derived LVEF was statistically different, and at a two-year follow-up, the mean absolute difference in LVEF was 6.5 ± 2.4% between the two groups at two years. As a result of the longer follow-up, Assmus et al. also explored major adverse cardiac events and survival following AMI between the treatment and the control. Combined death, rehospitalization for heart failure, and infarction were significantly lower for the BMC-treated group (*p* = 0.009) [14].

Duan et al. allocated to one of the two groups, CABG only (18 patients), or CABG with BMMNC (bone marrow mononuclear cell) transplantation (24 patients). This study showed improvements in left ventricular remodeling by showing significant improvements in LVEDD (mm) 2.06 1.67–5.61 0.000 LVESD (mm) 4.01 2.21–10.11 0.000 LVEDV (mL) 10.83 6.79–39.16 0.003 LVESV (mL) 11.39 6.45–32.51 0.001 LVEDVI (mL/m^2^) 6.59 4.42–20.27 0.005 LVESVI (mL/m^2^) 6.82 5.85–18.76 0.002 LV-mass (g) 8.61 6.36–36.65 0.008 LV-mass (g/m^2^) [18].

Choudhry et al. allocated 13 patients to receive intracoronary or intramyocardial injection of bone marrow stem cells while 14 patients received intracoronary or intramyocardial injection of serum alone. For the group of patients who received BMSCs, Tβ4 levels were significantly higher on D7 (after intracardiac injection) compared with D6 (*p* = 0.0461 *). Tβ4 is a protein implicated in regeneration. This study demonstrated this by showing that the group that demonstrated an improvement in NYHA symptom class (responders) showed a significantly greater increase in Tβ4 levels after cell reinfusion than the nonresponder group, *p* = 0.0126 * [17].

Gu et al. examined three different treatment arms which included the following: Group R (*n* = 15) which received a repeated intracoronary infusion of PBSC (peripheral blood stem cells) and one dose of G-CSF; Group S (*n* = 15) which received a single infusion of PBSC and a G-CSF dose; and Group C (*n* = 15) which received neither PBSC nor a G-CSF dose. All the patients underwent 12-month follow-up. LVEF in Group R (47.00 ± 4.90%) was significantly higher than that in Group S (44.40 ± 3.87%, *p* < 0.01) and Group C (40.80 ± 3.41%, *p* < 0.01) [20].

Bocchi et al. prospectively studied bone marrow stem cell therapy in 24 patients with non-ischemic refractory heart failure and compared this to a control consisting of 17 patients with heart failure. Additionally, BMSC patients randomly underwent granulocyte colony-stimulating factor (G-CSF) administration (14 patients) or G-CSF associated to BMSC intracoronary infusion (eight patients). After the first month, all BMSC patients received G-CSF with a one-month interval between each one. CD34+ cell peaks (per mm^3^) in BMSC patients were 19 ± 12 and in normal control 60 ± 20 (*p* = 0.003). In BMSC patients, after the 1st G-CSF left ventricular (LV) ejection fraction (EF) increased from 21.4 ± 4.7% to 23.6 ± 7.7% (*p* = 0.048), peak VO2 (mL/kg/min) from 9.9 ± 2.4 to 11.6 ± 3 (*p* = 0.04), functional class and quality of life improved compared to the HF control group where LVEF, RFEF and functional class were unchanged. Moreover, this study also indicated G-CSF administration alone also resulted in improvements in LVEF (4th G-CSF infusion increasing LVEF from 21.4% ± 4.7 to 28.5 ± 7.8%, *p* = 0.001 in all patients). This indicates CD34+ cell mobilization is impaired in HF, hence the additive beneficial effects of G-CSF infusion in addition to the therapeutic effects of intracoronary infusion of BMSC [19].

Table 3 elucidates some of the major adverse cardiovascular events that were seen in the studies included within this paper. For instance, Manginas et al. indicated that the delivery of hematopoetic stem cells was associated with a higher degree of restenosis and exacerbation of heart failure outcomes. Assmus et al., on the other hand, indicated that patients receiving HSC treatment had lower risk of major adverse cardiovascular events indicated in the table. De Rosa et al. applied a different approach where they summarized variations in MACE based upon the etiology of heart failure.

Table 4 summarizes the comorbidities of patients included within the study. These include hypertension, hyperlipidemia, diabetes mellitus, smoking, and coronary artery disease.

## 4. Discussion

Prior reviews have assessed the utility of mesenchymal stem cells for treating heart failure. In particular, these meta-analyses found that mesenchymal stem cells were able to improve LVEF in ischemic cardiomyopathy and improved cardiovascular outcomes following an ischemic event [21,22]. This is the first systematic review synthesizing the existing evidence on the potential of using HSCs therapeutically for heart failure.

Recognizing the therapeutic potential of HSCs in addressing heart failure is crucial, especially considering the limited regenerative capacity of the heart and the global susceptibility of millions to heart failure. Our systematic review reveals that HSCs can lead to improved cardiac health and functional outcomes such as EF, Tβ4, VO2max, and NYHA scale. In all studies that assessed ejection fraction (EF), every study indicated an improvement following HSC transplantation, with five out of seven studies reporting statistically significant changes in LVEF [13,15,16,18,19]. Although Choudry et al. did not evaluate changes in EF, they observed a statistically significant increase in Tβ4 levels following HSC transplantation [17]. Tβ4 is a polypeptide that is involved in cardiac repair through wound healing, anti-inflammatory effects, angiogenesis, and myocardial development. Tβ4 is thought to play a key role in facilitating the positive effects of cell therapy on cardiac function and enhancing EF [23]. Additionally, Zhou et al. elucidated that HSC regulate their perivascular niche by secreting angiopoietin-1 which subsequently mediates blood vessel maturity, stability and embryonic heart development [24]. This may suggest that HSCs are beneficial in the setting of HF through an angiogenesis mechanism, improving myocardial perfusion and promoting myocardial regeneration.

Our review presents several strengths. The majority of the included studies (71.43%) were RCTs with control groups [14,16,17,18,19]. Furthermore, studies had large sample sizes with a total of 1167 patients included in the current analysis. Studies implemented comprehensive follow-up protocols, with all but one of them tracking patients for a duration of one year or longer. This strengthens the quality of evidence for the two included prospective cohort studies. Finally, the analyzed studies gather high quality data from several countries on different continents. This allows our review to understand how diverse demographics and pathophysiological etiologies for heart failure may affect HSC treatment response.

Several limitations in our study are present due to differences in HSC administration, sample patient populations with respect to inclusion criteria, and cardiac function outcomes measured. Each study administered HSCs differently which made it difficult to compare studies on cell therapy outcomes. Furthermore, the site of cell administration differed across the analyzed studies, with some employing intracoronary injections [20] and others opting for intramyocardial injections [20]. Perin et al., in their canine model of acute myocardial infarction, illustrated that the method of stem cell delivery influences factors such as cell retention rate, survival, integration in the host, and functionality [25]. There is also evidence suggesting that, within mice models, intramyocardial routes of cell administration have a higher risk of arrhythmia occurrence in comparison to intracoronary routes [26]. Consequently, the method of delivery may impact the therapeutic outcomes of stem cell therapy for heart failure and therefore the consolidated results in our review. Furthermore, a significant source of confounding arises from the fact that some studies administered a combination of both hematopoietic and non-hematopoietic stem cells [14,15,16,17]. This complicates the assessment of whether improvements in cardiac function are genuinely attributed to HSCs or are instead influenced or produced by a different stem cell type. Finally, there are inconsistencies in the strategy used to identify or isolate HSCs between the analyzed studies. For example, some studies used CD33+, CD34+ progenitor cells or bone marrow mononuclear stem cells [13,18,19,20] while others focused on isolating bone marrow stem cells in general [16,17]. While CD34+ and bone marrow mononuclear stem cells contain only HSCs, bone marrow stem cells contain a mix of HSCs and mesenchymal stem cells [27]. This introduces potential confounding factors, making it challenging to determine whether improvements in cardiac function can be attributed specifically to HSCs.

Additionally, studies used different patient populations with distinct comorbidities (Table 4), which could have influenced the response to cell therapy. Type 2 diabetes mellitus (T2DM) is a prevalent comorbidity among patients in this review; however, there was variation among studies with respect to the proportion of samples comprising individuals with diabetes [14,15,16,17]. Rennert et al. demonstrated that diabetes may impair HSCs mobilization through remodeling the HSC niche required for differentiation [28]. Further, Kim et al. found that the angiogenic capability of mesenchymal stem cells was compromised within diabetic mouse models compared to healthy mice controls [29]. This compromises the potential of mesenchymal cells to improve tissue ischemia through neovascularization. Since HSCs can differentiate into mesenchymal stem cells, diabetes may impair the cell therapy’s ability to heal myocardial ischemia post MI [29]. In addition, only one study included patients that smoked cigarettes [20]. Siggins et al. found that, within mice models, cigarette smoke also alters the HSC niche and reduces the ability of HSCs to differentiate into MSCs [30]. However, it is interesting to note that evidence suggests that coronary artery bypass grafting (CABG), performed on all patients in the study by Duan et al., does not have an impact on stem cell mobilization [31].

Another limitation is that seven of eight included studies examine HSC transplantation within ischemic HF patients [13,14,16,17,18,19,20]. Even within the single study that examined non-ischemic HF, only 11.2% of patients had non-ischemic heart failure [15]. Similarly, seven of eight studies examined HFrEF with only 16.3% of patients presenting with HfpEF within the one study [15]. Therefore, it is difficult to generalize our finding that HSC improves cardiac function in nonischemic heart failure and HFpEF patient populations. Furthermore, the samples exhibited a disproportionate sex distribution, with a higher representation of males compared to females. There is considerable evidence showing that sex hormone differences between men and women influence HSC differentiation [32]. Therefore, our study may not be able to confidently generalize our findings to heart failure patients who are female.

Furthermore, a significant limitation arises from the presence of missing data in follow-up. While all studies conducted thorough follow-up with respect to EF, the studies did not collect follow-up data on other variables such as NT-pro-BNP or NYHA class. To fully appreciate the potential of HSCs to improve cardiac function in HF, it is important to corroborate EF values with other numerical measures and assessments of improvement.

The next steps to further explore and characterize the role of HSCs in the treatment of HF should involve aspects of increased demographic inclusion and more thorough evaluations of HF subtypes. We suggest larger multicenter randomized controlled trials with greater female representation. A larger sample size will improve external validity, and an increased proportion of females will better elucidate sex-based differences in HSC treatment, should any differences exist. Additionally, such studies should compare the efficacy and validity of HSC therapy across both ischemic and non-ischemic forms of HF, as well as HFpEF. Moreover, it is important to also evaluate BMNSCs with different antigen types, such as CD33+ and CD34+. Further studies should also evaluate a more diverse set of markers for improvement in HF, given that changes in ejection fraction may not be relevant for certain subtypes of HF (such as HFpEF or right sided HF). Future research should investigate the combined use of HSCs with established pharmacological and interventional therapies for heart failure (HF). It is essential to determine the indications and contraindications of HSCs in conjunction with these therapies. Considering that the proposed mechanisms of HSCs for cardiac regeneration operate independently of ACEi/ARNi/ARB, beta-blocker, MRA, and SGLT2i, pharmacological actions as well as ICD and CRT interventions, we hypothesize that HSC therapy will serve as an effective adjuvant treatment for HF when used alongside all classes of AHA-recommended therapies [33]. By broadening the scope of investigation to encompass these additional measures, researchers can gain a more comprehensive understanding of the potential benefits and limitations of HSC therapy in the management of heart failure.

Stem cell-based therapies have been explored in treating ischemic heart disease, which is among the leading causes of death globally, with a lifetime risk of 24% [2]. One of the ethical issues in hematopoietic stem cell therapy is harvesting HSCs from donor patients. Previous research shows that donors do not hesitate to make donations, regardless of their relationship with the recipient [34,35]. However, in situations where the donor and recipient are related, they are often informed of their medical procedures by the same physician, which has potential implications for informed consent [36]. This can skew the donor’s decision-making process and lead to incomprehensive informed consent. Therefore, it is critical for the donor to discuss their donation process with a clinician who is not closely associated with the medical case in order to reduce bias. Furthermore, donor patients also need to be informed of any pain/discomfort they may experience in the process of harvesting HSCs. This is especially important for donor patients that are treated with granulocyte colony-stimulating factor injections before and during the donation of HSCs. Studies have shown that donors experienced distress due to their lack of awareness of the bone pain caused by the granulocyte colony-stimulating factor pre-treatment [36]. Additionally, donor patients were also unaware of the potential physical pain/symptoms associated with transplantation, which led to donor patients experiencing anxiety. Therefore, it is critical that physicians provide a comprehensive and informed description of physical and mental symptom expectations in the donation process, without physician bias impacting this process.

## 5. Conclusions

Heart failure is one of the leading causes of morbidity, and the advent of stem cell therapy has the potential to help millions of patients globally. Currently, there is no recent review summarizing the efficacy of hematopoietic stem cells in treating heart failure. This review demonstrates that the administration of hematopoietic stem cells can lead to improved cardiac health and positive follow-up outcomes in EF, Tβ4, VO2max, and NYHA scale. However, more research is needed with greater female participant representation and larger sample sizes to generalize the review’s findings to the larger heart failure patient population. Further research needs to be directed towards analyzing HSC therapeutic potential in broader forms of cardiomyopathy beyond ischemic heart failure.

## Figures and Tables

**Table 1:** Population, intervention, comparison, and outcome (PICO) research strategy used.

Parameter	Description
Population (P)	Adult patients (age 18+) with heart failure
Intervention (I)	Hematopoietic stem cell therapy
Comparison (C)	Hematopoietic stem cell therapy or placebo or pre-therapy (baseline) measures or post-therapy measures
Outcome (O)	Peak VO2, New York Heart Association (NYHA) symptom class, major adverse cardiovascular events, ejection fraction, duration of follow-up appointments

**Table 2:** Study and participant characteristics.

Study	Country	Study Design	Group	Sample Size	Follow-Up	Age *	% Male	Etiology	Ejection Fraction *
[13]	Greece	Prospective cohort study	Total	24	NR			Ischemic HF	
			Treatment	12		50.1 ± 8.5	91.66		27.2 ± 6.8
			Control	12		64.8 ± 10.8	91.66		33.9 ± 69.1
[14]	Germany	RCT	Total	204	24 months	56 ± 11	82	Ischemic HF	
			Treatment	103		57 ± 11	NR		45.4 ± 9.4
			Control	101		55 ± 11	NR		48.7 ± 10.4
[15]	Germany	Retrospective cohort study	Total	775	1 month				
			AMI	126		54 ± 11	93.65	AMI	49 ± 10
			Chronic HF	562		62 ± 11	88.26	Ischemic HF	37 ± 11
			Chronic HF	87		57 ± 14	75.86	Non-ischemic HF	31 ± 11
[16]	UK	RCT	Total	9	12 months			Ischemic HF	
			Treatment	5		59 ± 11	80		30.5 ± 11.9
			Control	4		58 ± 4	100		27.8 ± 10.1
[17]	UK	RCT	Total	27	NR			Ischemic HF	
			Intramyocardial BMSC group	8		67.1 ± 10.5	100		32.0 ± 9.1
			Intracoronary serum group	8		59.3 ± 12.2	100		32.4 ± 8.3
			BMSC Group	5		62.0 ± 8.9	100		30.1 ± 3.4
			Serum Group	6		63.8 ± 6.6	100		27.6 ± 10.9
[18]	China	RCT	Total	42	12 months			Ischemic HF	
			CABG	18		56.56 ± 9.09	96		NR
			CABG + BMMNC	24		57.88 ± 8.52	94.4		NR
[19]	Brazil	RCT	Total	22	<1190 days	NR	NR	NR	31.4 ± 10
			G-CSF (control)	14		NR	NR	NR	29 ± 6.9
			BMSC	8		NR	NR	NR	35.8 ± 11.8

* Values reported as mean ± SD. NR = Not reported.

**Table 3:** Major adverse cardiovascular events (MACE).

Study	MACE
[13]	HF deterioration: 25%; stent re-stenosis 8.22%;
[14]	Cardiac death—Placebo/control: 5%; Treatment: 3%. Myocardial infarction—Placebo/control: 7%; Treatment: 0%. Revascularization—Placebo/control: 37%; Treatment: 25%. Documented ventricular arrhythmia—Placebo/control: 5%; Treatment: 6%. Stroke—Placebo/control: 2%; Treatment: 1%.
[15]	ICM: Native vessel-related complications: 4/455 dissection (nonflow-limitating), 1/455 main vessel occlusion, 1/455 side vessel occlusion, 1/455 thrombus formation/embolization, 2/455 arrhythmmia. Arterial graft-related complications: dissection (nonflow-limitating) 3/47. Venous graft-related complications: 1/60 arrhythmia DCM: 1/87 arrhythmiam 1/87 stroke, 6/87 repeat myocardial infarction, 4/87 deaths AMI: Procedural Complications Related to the Sole cell Administration Procedure: 1/126 dissection, 1/126 side branch occlusion, 2/126 thrombus formation.
[16]	NR
[17]	NR
[18]	CABG: 1/18 died
[19]	NR

NR = Not reported.

**Table 4:** Percent of patients with co-morbidities.

Study	Group	HTN %	Hyperlipidemia %	Diabetes %	Smoking %	CAD %
[14]	Total	NR	NR	NR	NR	NR
	Treatment group	54	52	12	NR	NR
	Control group	60	59	21	NR	NR
[15]	AMI	NR	51	26		100
	ICM	NR	82	32	73	100
	DCM	NR	53	18	68	100
[16]	Total	NR	NR	NR	NR	NR
	Patients receiving intracoronary infusion of BMSC	NR	NR	40	NR	NR
	Patients receiving intracoronary infusion of serum only	NR	NR	0	NR	NR
[17]	Total	NR	NR	NR	NR	NR
	Intramyocardial BMSC group	NR	NR	37.5	NR	NR
	Intracoronary serum group	NR	NR	25	NR	NR
	Intracoronary BMSC group	NR	NR	20		
	Intracoronary serum group	NR	NR	33.3	NR	NR
[18]	Total	NR	NR	NR	NR	NR
	CABG	11.1	NR	NR	NR	NR
	CABG + BMMNC	16.7	NR	NR	NR	NR

NR = Not reported.

## Data Availability

Not applicable.

## Abbreviations

HSC	Hematopoietic Stem Cells
MSC	Mesenchymal Stem Cells
NYHA	New York Heart Association
VEGF	Vascular Endothelial Growth Factor
BNP	B-type Natriuretic Peptide
EF	Ejection Fraction
BMSC	Bone Marrow Stem Cell
AMI	Acute Myocardial Infarction
BMC	Bone Marrow-derived progenitor Cells
BMMNC	Bone Marrow Mononuclear Cell
PBSC	Peripheral Blood Stem Cells
G-CSF	Granulocyte Colony-Stimulating Factor
LVEF	Left Ventricular Ejection Fraction
RCT	Randomized Controlled Trials
CD33	Cluster of Differentiation 33
CD34	Cluster of Differentiation 33
T2DM	Type 2 Diabetes Mellitus
CABG	Coronary Artery Bypass Grafting
HF	Heart Failure
AMI	Acute Myocardial Infarction
BMC	Bone Marrow-derived progenitor Cells
BMMNC	Bone Marrow Mononuclear Cell
BMSC	Bone Marrow Stem Cell
BNP	B-type Natriuretic Peptide
CABG	Coronary Artery Bypass Grafting
CD33	Cluster of Differentiation 33
CD34	Cluster of Differentiation 33
EF	Ejection Fraction
G-CSF	Granulocyte Colony-Stimulating Factor
HF	Heart Failure
HSC	Hematopoietic Stem Cells
LVEF	Left Ventricular Ejection Fraction
MSC	Mesenchymal Stem Cells
NYHA	New York Heart Association
PBSC	Peripheral Blood Stem Cells
RCT	Randomized Controlled Trials
T2DM	Type 2 Diabetes Mellitus
VEGF	Vascular Endothelial Growth Factor
